# Reduced Fecal Calprotectin and Inflammation in a Murine Model of Atopic Dermatitis Following Probiotic Treatment

**DOI:** 10.3390/ijms21113968

**Published:** 2020-05-31

**Authors:** Myoung-Ju Kim, Ji-Young Kim, Minje Kang, Moo-Ho Won, Seok-Ho Hong, Young Her

**Affiliations:** 1 Department of Internal Medicine, School of Medicine, Kangon National University, Chuncheon 24341, Korea; unknowman@naver.com (M.-J.K.); jeans330910@gmail.com (J.-Y.K.); 2 Division of Biomedical Convergence, Department of Biomedical Sciences, Kangwon National University, Chuncheon 24341, Korea; hobe0@naver.com; 3 Department of Neurobiology, School of Medicine, Kangwon National University, Chuncheon 24341, Korea; mhwon@kangwon.ac.kr; 4 Department of Dermatology, School of Medicine, Kangwon National University, Kangwon National University Hospital, Chuncheon 24341, Korea

**Keywords:** atopy, calprotectin, probiotics, inflammation

## Abstract

Atopic dermatitis (AD) is one of the most common skin diseases with inflammation, chronic relapses, and intense pruritus. Its pathogenesis includes genetic susceptibility, an abnormal epidermal lipid barrier, and an increased production of IgE due to immune dysregulation. Recently, AD has been reported to be associated with intestinal inflammation and dysbiosis in human and murine models. Various probiotics are being used to control intestinal dysbiosis and inflammatory reactions. However, it is difficult to predict or determine the therapeutic effects of the probiotics, since it is rare for clinicians to use the probiotics alone to treat AD. It is also difficult to check whether the intestinal inflammation in patients with AD has improved since probiotic treatment. The aim of the present study was to determine whether mice with induced atopic dermatitis had any changes in fecal calprotectin, an indicator of intestinal inflammation, after probiotic administration. Our results showed that the fecal calprotectin levels in mice with induced dermatitis decreased significantly after the administration of probiotics. In addition, epidermal skin lesions were attenuated and inflammatory-related cytokines were downregulated after the administration of probiotics in mice with induced dermatitis. These results suggest that changes in fecal calprotectin levels could be used to assess the effectiveness of a probiotic strain as an adjuvant treatment for AD.

## 1. Introduction

Atopic dermatitis (AD) is the most common chronic pruritic inflammatory dermatosis and affects 15–30% of children and 2–10% of adults [1,2,3]. AD’s etiology is not completely understood, but it is multifactorial. AD is characterized by complex interactions between genetic and environmental factors, such as skin barrier dysfunction, allergy/immunity, and pruritus [4,5]. Filaggrin is a key protein involved in skin barrier function. Th2 cytokines decrease filaggrin expression by keratinocytes [6,7]. They also promote the production of IgE to low doses of allergens, a characteristic commonly observed in AD [8]. Although biologics and immunosuppressants targeting immune cells and cytokines have good therapeutic effects on AD, they are not applicable to all patients because of high prices or side effects such as renal toxicity [9,10,11].

Although the direct causes and pathogenesis of AD are poorly defined, concurrent dysbiosis and the disruption of the intestinal epithelial barrier can cause secondary uncontrolled intestinal epithelial inflammation, which creates an environment that allows the easy penetration of allergens [12]. Since AD is associated with dysbiosis, probiotics may be useful for preventing and treating AD via microbial, epithelial, and immune effects [13]. Recently, probiotics using various combinations of strains have been released. Many scientists are interested in treating allergies using probiotics or byproducts [14,15]. However, the results of treatment with probiotics vary across patients, even when the same probiotics are used [15]. In addition, there are no markers to indicate that the epithelial inflammation of the gut in AD patients has been impacted after treatment with probiotics. Calprotectin, also known as S100A8/A9, MRP8/14, and calgranulin A/B, is a 36 kDa calcium and zinc binding protein belonging to the S100 family [16]. Persistent inflammatory reactions trigger increases in calprotectin levels [17]. Additionally, when the inflammatory response decreases, the levels of calprotectin in the plasma, urine, and feces decrease. Furthermore, increased calprotectin levels have been found in chronic inflammatory bowel disease and allergic diseases [18,19,20]. Taken together, fecal calprotectin is an important clinical marker for gastrointestinal tract inflammation. In this regard, this study was conducted to determine if calprotectin could be used as an indicator of the downregulation of gut inflammation after the administration of probiotics in an AD-like skin model.

## 2. Results

### 2.1. Probiotics Can Improve AD-Like Skin Lesions in Oxazolone (Ox)-Induced AD

**Figure 1:**
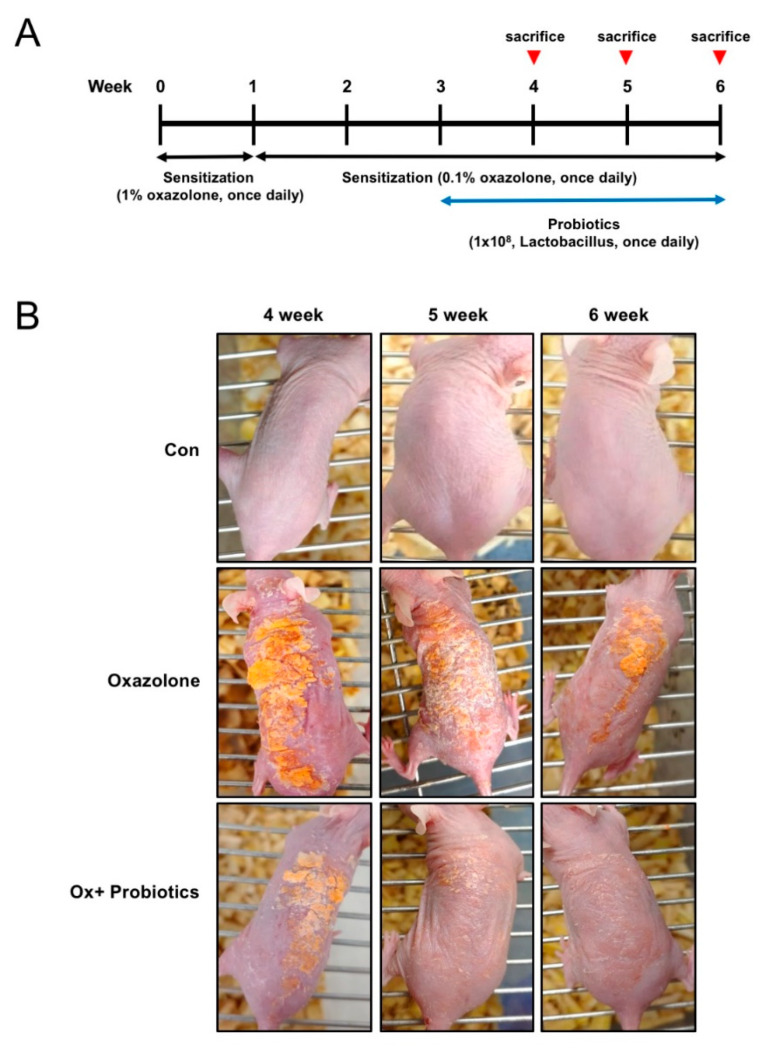
Effects of probiotics on the development of an Ox-induced atopic dermatitis (AD) mouse model. (**A**) Schematic representation of the experiment. Development of Ox-induced AD mouse model with multiple features of AD. (**B**) Clinical features of AD skin lesions. Con, control mice; Ox, oxazolone; Ox + Probiotics, Ox-induced AD mice with probiotics treatment. Two independent experiments were performed, and at least three mice per group were used in each experiment.

To study the effects of probiotics on AD, we established a mouse model of AD-like skin lesions by using mice treated with Ox. The mice were sensitized with Ox for 6 weeks (1% for 1 week followed by 0.1% for 5 weeks). Probiotics (1 × 10^8^) were orally administered to Ox-induced AD mice daily for 3 weeks (from Week 3 to Week 6) in parallel with the sensitization with 0.1% Ox (Figure 1A). We found that the dorsal skin of Ox-treated mice at 3 weeks exhibited AD symptoms including itching, severe dryness, erythema, and erosion. Interestingly, increases in atopic symptoms in Ox-treated mice were significantly reduced from one week after the administration of probiotics. (Figure 1B). These results suggest that sustained stimulation such as with Ox can induce AD-like skin lesions, which were alleviated by treatment with probiotics.

### 2.2. Probiotics Can Attenuate Epidermal Skin Lesions in Ox-Induced AD Mice

**Figure 2:**
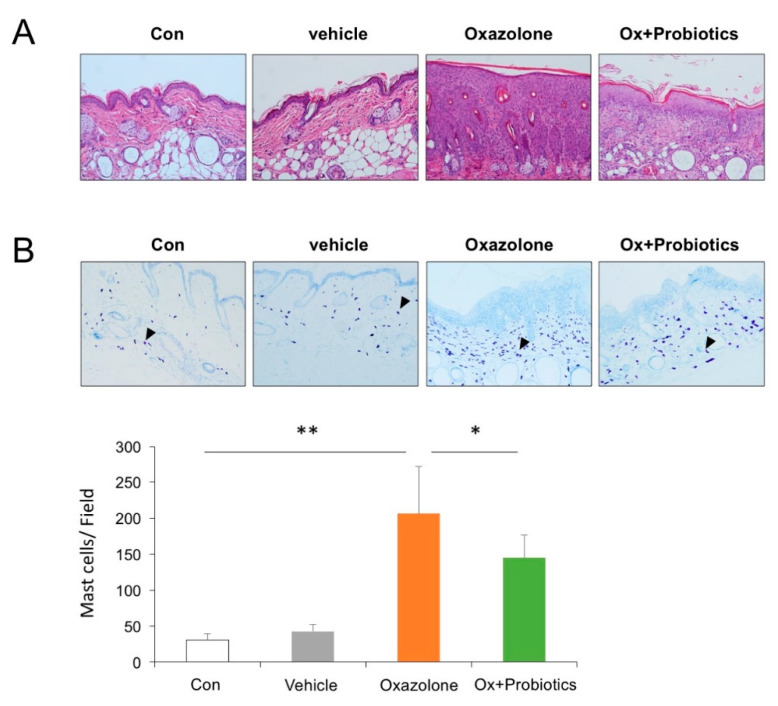
Effects of probiotics on the epidermal skin lesions in Ox-induced AD mice. (**A**) Histological features of epidermal skin lesions. Tissues were fixed in 10% formaldehyde, embedded in paraffin, and sectioned. The sections were stained with H&E (magnification, 400×) (**B**) Toluidine blue staining to detect infiltrating mast cells in the dorsal skin of mice. Arrowheads indicates mast cells (magnification, 400×). Data pooled from two independent experiments are presented as the mean ± SD. * *p* < 0.05, ** *p* < 0.01.

We explored the effects of probiotics on the epidermal skin lesions of Ox-induced AD mice by the microscopic analysis of H&E- and toluidine blue-stained sections of epidermal skin. Ox-induced AD mice exhibited typical characteristics of AD, including parakeratosis, hyperkeratosis, and the infiltration of inflammatory cells (Figure 2A). In addition, the number of mast cells was dramatically increased in the Ox-induced AD mice compared to that in the control mice (Figure 2B). However, in skin sections of the Ox-induced AD mice treated with probiotics, the epidermal tissue was significantly thinner and the number of mast cells as well as the infiltration of inflammatory cells was reduced in the dermis (Figure 2A,B). These data supported the idea that probiotics could improve the skin symptoms of Ox-induced AD mice by inhibiting the migration of inflammatory cells such as mast cells and lymphocytes, protecting the skin barrier against atopic inflammation.

### 2.3. Probiotics Can Reduce Th2-Associated Cytokines and Pro-Inflammatory Cytokines

**Figure 3:**
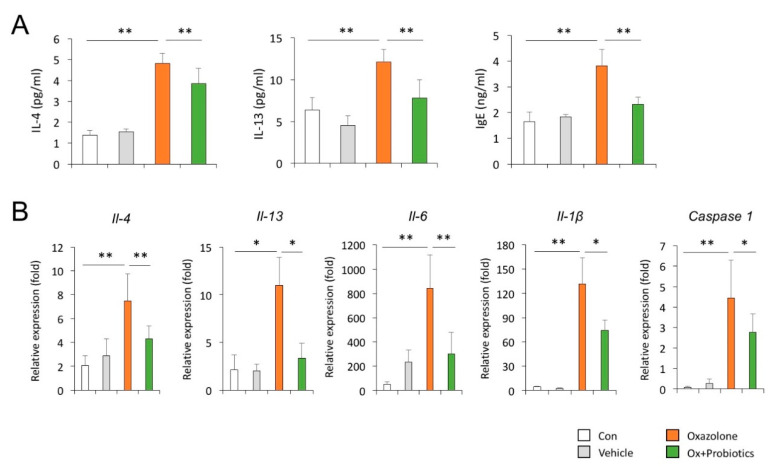
Anti-inflammatory effects of probiotics on the development of AD. (**A**) Serum total IgE, IL-4, and IL-13 levels were determined by ELISA. Con (*n* = 3), vehicle (*n* = 3), Ox (*n* = 5), Ox + probiotics (*n* = 5). (**B**) mRNA expression levels of inflammation-related genes were determined by qPCR in dorsal skin tissues. Con (*n* = 3), vehicle (*n* = 3), Ox (*n* = 5), Ox + Probiotics (*n* = 5). Data pooled from two independent experiments are presented as the mean ± SD. * *p* < 0.05, ** *p* <0.01.

To investigate the effects of probiotics on the secretion of Th2-associated cytokines in the development of AD, the expression levels of the cytokines were measured in the serum and skin of an Ox-induced AD mouse model. As expected, the secretory levels of IL-4, IL-13, and IgE were significantly increased in the serum of Ox-induced AD mice compared to those in the control mice (Figure 3A). The transcript levels of *Il-4* and *Il-13* were also markedly elevated in the skin tissue of Ox-induced AD mice compared to those in the control mice (Figure 3B). Furthermore, qPCR demonstrated that the increased secretion and transcript levels of the Th2-associated cytokines in the Ox-induced AD mice were significantly reduced by probiotic treatment (Figure 3A,B). We further determined the effects of probiotics on the expression of pro-inflammatory cytokines in the skin of Ox-induced AD mice. We found that probiotic treatment effectively attenuated the elevated expression of *Il-6*, *Il-1β* and *Caspase-1* transcripts in the dorsal skin of AD mice (Figure 3B). These data indicate that the accumulation of inflammatory cells in Ox-induced AD mice is induced by the production of inflammatory cytokines, whereas the administration of probiotics to AD mice can regulate inflammatory cell-mediated allergic responses and inflammation, possibly by downregulating the production of these cytokines.

### 2.4. Probiotics Can Effectively Restore Impaired Skin Barrier Formation

**Figure 4:**
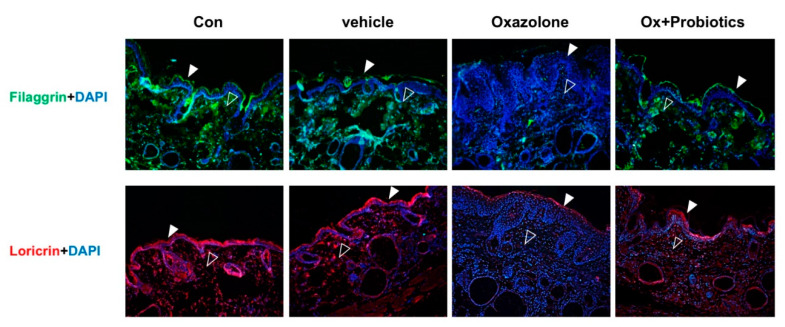
Effects of probiotics on skin barrier dysfunction in the development of AD. Paraffin-embedded skin tissues were stained with anti-filaggrin (green) and anti-loricrin (red). All sections were counterstained with 4′,6-diamidino-2-phenylindone (DAPI) (blue). Closed arrowheads indicate epidermis. Open arrowheads indicate dermis (magnification, ×400). Two independent experiments were performed, and at least three mice per group were used in each experiment.

To investigate whether probiotics might have skin barrier-recovering effects, we examined the expression of skin barrier proteins such as loricrin and filaggrin, known to contribute to skin barrier function and epidermal hydration. Immunofluorescence staining showed that the signal intensities of filaggrin and loricrin were decreased in both the epidermis and dermis in Ox-induced AD mice compared to those in control mice, whereas mice administered probiotics showed significant increases in the expression of these proteins (Figure 4). These results suggested that Ox applied to mouse skin could lead to the development of skin barrier dysfunction, whereas probiotics could prevent skin barrier destruction.

### 2.5. Probiotics Can Decrease the Level of Calprotectin Increased in the Feces of AD Mice

**Figure 5:**
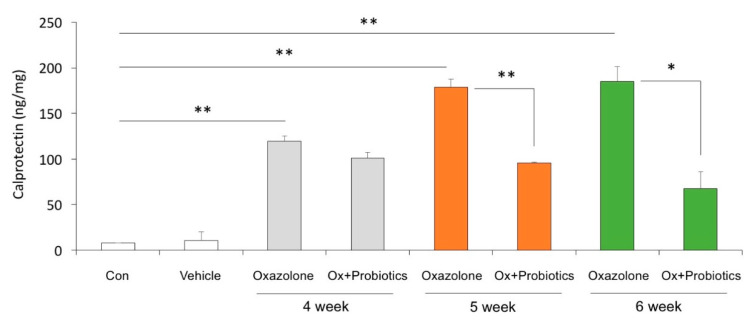
Effects of probiotics on calprotectin levels in Ox-induced AD. The levels of fecal calprotectin in stool samples of the Con, Ox, and Ox + Probiotics groups. The concentrations of calprotectin were measured by ELISA. The stool samples were collected once a week for 3 weeks after the administration of probiotics. Data pooled from two independent experiments are presented as the mean ± SD. * *p* < 0.05, ** *p* < 0.01.

Fecal calprotectin is a protein abundant in the cytoplasm of neutrophils and monocytes. Consequently, the calprotectin level is increased in inflammatory processes such as chronic inflammatory bowel disease and allergic disease [18,19,20]. To demonstrate the intestinal inflammatory control effect of probiotics in the Ox-induced AD mice model, we measured calprotectin levels in the feces by ELISA. Calprotectin levels were significantly elevated from one week after sensitization in the Ox-induced AD mice up to 6 weeks, the end of the experiment. Surprisingly, the level of calprotectin that increased in the Ox-induced AD mice was markedly decreased in the mice administered probiotics (Figure 5). These findings indicate that probiotics can decrease the levels of calprotectin seen in Ox-induced AD.

**Figure 6:**
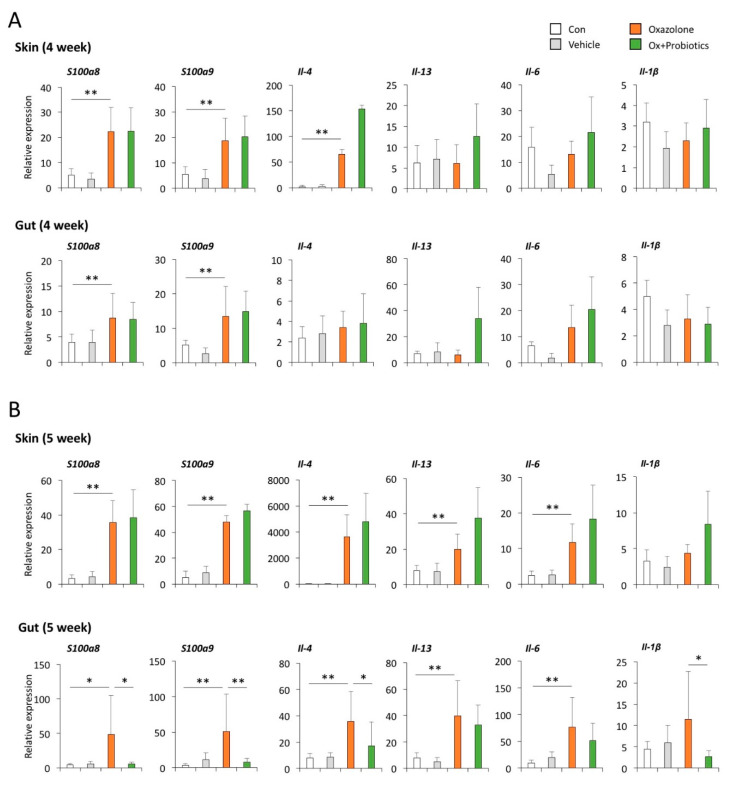
Effects of probiotics on the temporal expression of inflammatory cytokines and calprotectin in the skin and guts of Ox-induced AD mice. (**A**) Transcript levels of calprotectin (*S100a8* and *S100a9*) and inflammatory cytokines in the skin and guts of AD mice treated with probiotics for 1 week (from Week 3 to 4) in parallel with the sensitization with 0.1% Ox. Bars indicate the means ± SD. * *p* < 0.05, ** *p* < 0.01. (**B**) Transcript levels of calprotectin and inflammatory cytokines in the skin and guts of AD mice treated with probiotics for 2 weeks (from Week 3 to 5) in parallel with the sensitization with 0.1% Ox. Data pooled from two independent experiments are presented as the mean ± SD. * *p* < 0.05, ** *p* < 0.01.

We further asked if calprotectin was detectable in the dorsal skin of mice and determined the temporal expression of calprotectin transcripts (*S100a8* and *S100a9*) in the skin and gut of Ox-induced AD mice with and without probiotic treatment. We found that both the transcript and protein of calprotectin (*S100a8* and *S100a9*) were detectable in the skin of mice and more abundantly expressed in Ox-induced AD mice (Figure 6 and Figure S1). However, the elevated levels of the calprotectin transcript and protein in the dorsal skin of Ox-induced AD mice were not reduced by probiotic treatment for 1 and 2 weeks, respectively (Figure 6). We also found that the levels of calprotectin and inflammatory cytokines, increased in the guts of Ox-induced AD mice, were significantly decreased in mice administered with probiotics for 2 weeks, even though there was no effect of probiotic treatment on the reduction of calprotectin and inflammatory cytokines in the skin of the Ox-induced AD mice (Figure 6B and Figure S1). Together, these findings suggest that calprotectin might be an effective indicator of improvements in intestinal inflammation following the administration of probiotics as an adjuvant therapy for atopic patients.

## 3. Discussion

In this study, we found that the levels of fecal calprotectin were decreased significantly, accompanied by clinical and immunological improvement, in an Ox-induced AD mouse model following the administration of probiotics. Many studies have shown that after the administration of probiotics, the severity of atopic dermatitis decreases, immunological changes in the serum are detected, and there is a temporary intestinal microbiome change. These changes are the result of the reduction in inflammation in the intestine through probiotics, and through this, the effect of allergen invasion is reduced. However, to date, there has been no research on whether probiotics actually improve inflammation in the intestine.

As initial barriers to an external environment, the intestinal tract and skin are essential for maintaining physiological homeostasis [5]. Furthermore, gut health is linked to skin homeostasis and allostasis [14,15]. The intestinal microbiome can prevent the invasion of exogenous allergens both directly (by competitively binding to epithelial cells) and indirectly (by triggering immunoprotective responses) [8]. For immunoprotection, intestinal immunomodulatory metabolites, especially short-chain fatty acids (SCFAs), produced by the intestinal microbiome are essential. SCFAs are known for their anti-inflammatory actions mediated by G-protein coupled receptor 43 and for their contribution to epithelial barrier integrity [9,16]. However, the low-fiber and high-fat contents of the western diet can alter the gut microbiome, resulting in the deficient production of SCFAs [9,16]. For example, a metagenomic analysis of fecal samples from patients with AD has demonstrated a significant reduction in *Faecalibaterium prausnitzii* compared to in control patients [10]. In parallel, a decrease in SCFA production was also observed in AD patients. The authors cited a putative positive feedback loop involving intestinal dysbiosis in association with *F. prausnitzii* and epithelial barrier disruption, secondary to uncontrolled inflammation. As such, intestinal dysbiosis, in the form of an unbalanced bacterial composition or aberrant immune reaction to commensal flora, can result in intestinal inflammation and altered gut permeability to allergens, which in turn can affect the development and severity of allergic disease [14,15,16].

Given the integral role of the gut microbiome in the development of the immune system and homeostasis, probiotics might be useful for modifying the microbial composition and improving impaired barrier function via the production of SCFAs. Thus, the use of probiotics in AD should ultimately correct immunological imbalance, reduce intestinal inflammation, and reduce the invasion of allergens. Many studies show they can promote immunologic balance by inhibiting proinflammatory cytokines (interleukin IL-4, interferon gamma, and IL-17) and promoting regulatory cytokines (IL-10 and transforming growth factor beta). Probiotics can also increase the expression of regulatory T cells, which can migrate to the skin and inhibit Th2 and Th17 responses. Thus, probiotics may have a therapeutic role in AD, in addition to their preventative role [17,18,19]. Numerous studies have demonstrated good clinical outcomes following treatment with probiotics, including improved dermatitis and reduced itching in atopic animal models and patients, as well as the restoration of immunological balance [6,20]. Of the various strains used experimentally and clinically, *Lactobacillus* species are the most commonly tested probiotics in the context of AD treatment [20,21]. In the present study, the administration of *Lactobacillus* in an atopic animal model resulted in positive immunological changes and clinical improvement, similar to the results of previous reports [6,20,21,22,23]. Many studies have shown that the severity of dermatitis and the immunological marker profile are improved after probiotic treatment [21,22,23]. In this study, as in past studies, the clinical symptoms of mice improved and reductions in IgE and Th2 cytokines, IL-4 and IL-13, confirmed an improvement of the immunological imbalance.

Calprotectin, an antimicrobial protein released via neutrophil activation or monocyte–endothelial cell adhesion, can be used as a marker of intestinal inflammation [7]. Fecal calprotectin levels correlate positively with neutrophil migration into the gut lumen [24]. It can serve as a candidate biomarker for diagnosis and follow-up as well as a predictive indicator of therapeutic responses in inflammation-associated diseases [11]. In patients with inflammatory bowel disease, the level of fecal calprotectin is closely related to mucosal gastrointestinal inflammation [25]. Thus, fecal calprotectin is considered a sensitive marker of mucosal healing in gut inflammation [26]. In studies of the link between intestinal inflammation and allergic diseases, calprotectin is rarely used. A recent study has shown that fecal calprotectin levels are higher in children with a non-IgE-mediated cow’s milk protein allergy than in controls [12]. In addition, high fecal calprotectin levels have been found in children with an egg allergy [27]. Orivuori et al. [13] have demonstrated that infants with high fecal calprotectin levels at the age of 2 months have an increased risk of developing AD by age 6. Seo et al. [26] have reported that elevated fecal calprotectin levels are associated with the severity of AD in children. Probiotics can be administered to promote the diversity of gut microbiome and suppress inflammation. However, few studies have actually assessed the ability of probiotics to inhibit the gut inflammation that facilitates the invasion of antigens. In inflammatory bowel disease, a decrease in the fecal calprotectin level following the administration of probiotics for 4 weeks indicates that probiotics may affect the host by supplying beneficial intestinal bacteria [28,29,30]. Interestingly, infants with low fecal calprotectin levels showed a low abundance of *Lactobacillaceae* and a high percentage of *Escherichia* in their feces, suggesting that the early microbial colonization of the gut could regulate intestinal inflammation [31,32]. This further implies that the administration of probiotics can diminish gastrointestinal inflammation and allergic responses. The purpose of administering probiotics to patients with atopy is to alter their intestinal flora, reduce intestinal inflammation and gut permeability (via SCFAs), and enhance immunological protection. It is impractical to perform intestinal biopsies in subjects with AD to assess whether adjuvant probiotic therapy has modulated intestinal inflammation in the context of AD. Thus, we speculated that we could follow probiotic-mediated changes in intestinal inflammation by tracking fecal calprotectin and that changes in intestinal fecal calprotectin levels would mirror changes in the levels of type 2 inflammatory cytokines in the serum. In this study, we found that the levels of fecal calprotectin were decreased and dermatitis was improved after the administration of probiotics for 4 weeks. Calprotectin can be quantified in a straightforward and non-invasive manner. Its test results can be confirmed quickly. Therefore, we propose that calprotectin can be used as an indicator of intestinal inflammation before and after treatment with probiotics as adjuvant therapy in atopic patients. However, this study neither confirmed whether calprotectin could serve as a marker of therapeutic efficacy using various probiotics nor confirmed whether the level of calprotectin changes with the dose of probiotics. In addition, the calprotectin levels in our mouse model were not measured before treatment, i.e., no cutoff was applied. Baseline differences might have affected the treatment outcomes. Thus, clinical research in humans using various probiotics is needed in the future to validate our findings.

## 4. Materials and Methods

### 4.1. Animals

SKH-1 hairless mice were purchased from Dooyeol Biotech. Female mice were housed in a specific pathogen-free facility. All animal procedures were approved by the Institutional Animal Care and Use Committee of Kangwon National University (KW-181129-2, 29 November 2018). The SKH-1 hairless mice were divided into following four groups: Group 1, the control group (*n* = 6); Group 2, the vehicle group (*n* = 6); Group 3, Ox-induced AD (*n* = 6); and Group 4, Ox-induced AD with probiotics (*n* = 6). The AD groups were sensitized with 1% Ox in acetone (Sigma-Aldrich, Burlington, MA, USA, E0753) daily for 1 week, while the vehicle group were treated with acetone only. After 1 week, the vehicle group or Ox-induced AD groups were treated with acetone or 0.1% Ox daily for 5 weeks, respectively. Probiotics (Lactobacillus, 1 × 10^8^ CFU in 500 μL per day; HANWHA PHARMA, 612091-5-2) were orally administered to Ox-induced AD mice with 0.1% Ox daily for 3 weeks (from 3 to 6 week) in parallel with the sensitization.

### 4.2. ELISA

Mouse IgE, IL-4, and IL-13 concentrations were determined in the serum by using the ELISA Kits (Finetest, Wuhan, China, #EM0211, #EM0119, #EM0103) according to the manufacturer’s protocols. For the detection of the fecal calprotectin level, fecal supernatant samples were lysed in RIPA (ELPIS, Seoul, Korea, #EBA-1140) with protease inhibitor (Thermo Scientific, Waltham, MA, USA, #78430) and analyzed with ELISA kits (Finetest, #EM1620).

### 4.3. Immunohistochemistry

The immobilized dermal skin sections were stained with hematoxylin and eosin (BBC, #3540, #3600). For the investigation of mast cell infiltration, sections were stained with toluidine blue O (Sigma-Aldrich, Burlington, MA, USA, #T3260-5G). To evaluate mast cell counts, five mice in each group were used and data were obtained from four fields and averaged.

### 4.4. Immunofluorescence Staining

The paraffin block-embedded skin sections were immobilized, and then immunofluorescence was performed on deparaffinized mouse skin tissue slides. The slides were blocked with 10% goat serum (normal goat serum, Cell Signaling, H5425) in distilled water for 1 h at room temperature. After washing, the tissue sections were incubated with primary antibodies against filaggrin (Santa Cruz, TX, USA, #sc66192) and loricrin (Abcam, Cambridge, UK, #ab85679) overnight at 4 °C. The slides were then incubated with corresponding secondary antibodies for 1 h at room temperature as follows: goat-anti mouse Alexa Fluor^®^ 488 (Abcam, #150077) and goat-anti mouse Alexa Fluor^®^ 594, (Abcam, #150116). All the sections were counterstained with DAPI.

### 4.5. Quantitative Real-Time PCR (qPCR)

Quantitative real-time PCR was performed as previously described [33]. Briefly, total RNA from dorsal skin was extracted using TRIzol reagent (Ambion, 15596018) and quantitative PCR analyses were performed using a Step One Plus real time PCR system (Applied Biosystems, Warrignton, UK) with all the reactions performed in triplicate. The qPCR reactions involved pre-denaturation at 95 °C for 10 min, 95 °C for 15 s, and 60 °C for 1 min for 40 cycles. The primers are listed in Table 1.

### 4.6. Western Blotting

Western blotting was performed as previously described [34]. Briefly, lysates from dorsal skin and gut tissues were lysed in RIPA with protease inhibitor and quantified using the BCA protein assay (Thermo Scientific, Waltham, MA, USA, 23225). Then, 20 μg of protein were loaded into an SDS-PAGE gel (10% or 12%) and transferred to PVDF membranes (Millipore, Burlington, MA, USA, IPVH00010). The nonspecific binding of proteins was blocked with 5% skim milk for 1 h at room temperature, then the membranes were incubated with the primary antibodies anti-S100A8 (R&D system, Minneapolis, MN, USA, af2059) and anti-S100A9 (R&D system, af2065) overnight at 4 °C. Their corresponding secondary antibodies were incubated with the membranes for 1 h at room temperature. The membranes were scanned with a ChemiDoc imaging system (Bio-Rad Laboratories).

### 4.7. Statistical Analysis

To evaluate the therapeutic effects of probiotics in vivo, at least three animals per group were used in each experiment. Data pooled from two independent experiments are presented as the mean ± standard deviation (SD) unless otherwise indicated. All data analysis was performed in the GraphPad Prism software (GraphPad Software Inc, San Diego, CA, USA). The non-parametric Mann–Whitney U test was used for data analyses, and a value of *p* < 0.05 was considered statistically significant.

## 5. Conclusions

Our results indicated the existence of anti-inflammatory effects of and attenuation of epidermal skin lesions by probiotics treatment in an Ox-induced AD mice model. The administration of probiotics significantly decreased the levels of fecal calprotectin in an Ox-induced AD mouse model. Overall, these results suggest that changes in fecal calprotectin levels could be used to assess the effectiveness of a probiotic strain as an adjuvant treatment for AD.

## Figures and Tables

**Table 1:** Primer sequences used for qPCR.

Genes		Sequences 5′ to 3′	Product Size (bp)
*ll-6*	F	AGGATACCACTCCCAACAGACCT	141
	R	CAAGTGCATCATCGTTGTTCATAC	
*ll-4*	F	AGATGGATGTGCCAAACGTCCTCA	107
	R	AATATGCGAAGCACCTTGGAAGCC	
*ll-13*	F	TGAGGAGCTGAGCAACATCACACA	176
	R	TGCGGTTACAGAGGCCATGCAAT	
*ll-10*	F	GCCAAGCCTTATCGGAAATG	102
	R	CACCCAGGGAATTCAAATGC	
*ll-1α*	F	CCGACCTCATTTTCTTCTGG	104
	R	GTGCACCCGACTTTGTTCTT	
*ll-1β*	F	CCCAAGCAATACCCAAAGAA	133
	R	GCTTGTGCTCTGCTTGTGAG	
*Caspase 1*	F	AGATGGCACATTTCCAGGAC	221
	R	GATCCTCCAGCAGCAACTTC	
*S100a8*	F	GCCCTCTACAAGAATGACTTCAAG	151
	R	ATC ACC ATC GCA AGG AAC TCC	
*S100a9*	F	TGGTGGAAGCACAGTTGGCAAC	165
	R	CAGCATCATACACTCCTCAAAGC	
*Gapdh*	F	GTTGTCTCCTGCGACTTCA	184
	R	GGTGGTCC GGGTTTCTTA	

## Supplementary Materials

Supplementary materials can be found at https://www.mdpi.com/1422-0067/21/11/3968/s1. Figure S1. Effects of probiotics on the temporal protein expression of calprotectin in the skin and guts of Ox-induced AD mice.
