# Supplementary material for: Reduced Fecal Calprotectin and Inflammation in a Murine Model of Atopic Dermatitis Following Probiotic Treatment
Source: Int J Mol Sci. 2020 May 31;21(11):3968. doi: 10.3390/ijms21113968 (PMC7312066; doi:10.3390/ijms21113968)
Supplement: Supplementary file 1 [file ijms-21-03968-s001.pdf]

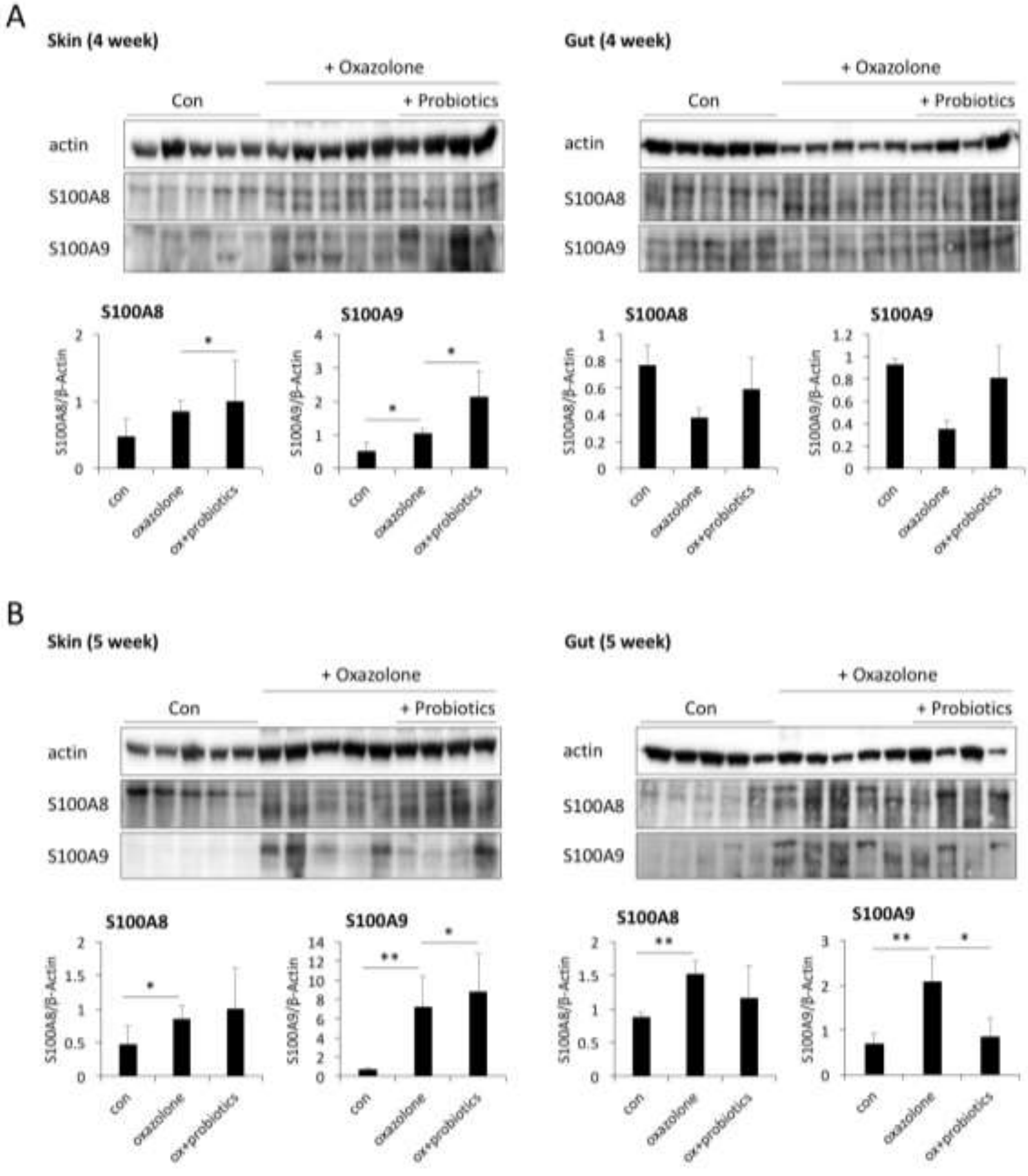

Figure S1. Effects of probiotics on the temporal protein expression of calprotectin in the skin and guts of Ox-induced AD mice. (**A**) Protein levels of calprotectin (S100a8 and S100a9) in the skin and guts of AD mice treated with probiotics for 1 week (from Week 3 to 4) in parallel with the sensitization with 0.1% Ox. Bars indicate the means ± SD. *$p < 0.05$. (**B**) Protein levels of calprotectin in the skin and guts of AD mice treated with probiotics for 2 weeks (from Week 3 to 5) in parallel with the sensitization with 0.1% Ox. Data pooled from two independent experiments are presented as the mean ± SD. * $p < 0.05$, ** $p < 0.01$.
